# Mutation scanning of peach floral genes

**DOI:** 10.1186/1471-2229-11-96

**Published:** 2011-05-23

**Authors:** Yihua Chen, H Dayton Wilde

**Affiliations:** 1 Horticulture Department, University of Georgia, Athens, GA 30602, USA

## Abstract

**Background:**

Mutation scanning technology has been used to develop crop species with improved traits. Modifications that improve screening throughput and sensitivity would facilitate the targeted mutation breeding of crops. Technical innovations for high-resolution melting (HRM) analysis are enabling the clinic-based screening for human disease gene polymorphism. We examined the application of two HRM modifications, COLD-PCR and QMC-PCR, to the mutation scanning of genes in peach, *Prunus persica*. The targeted genes were the putative floral regulators *PpAGAMOUS *and *PpTERMINAL FLOWER I*.

**Results:**

HRM analysis of *PpAG *and *PpTFL1 *coding regions in 36 peach cultivars found one polymorphic site in each gene. *PpTFL1 *and *PpAG *SNPs were used to examine approaches to increase HRM throughput. Cultivars with SNPs could be reliably detected in pools of twelve genotypes. COLD-PCR was found to increase the sensitivity of HRM analysis of pooled samples, but worked best with small amplicons. Examination of QMC-PCR demonstrated that primary PCR products for further analysis could be produced from variable levels of genomic DNA.

**Conclusions:**

Natural SNPs in exons of target peach genes were discovered by HRM analysis of cultivars from a southeastern US breeding program. For detecting natural or induced SNPs in larger populations, HRM efficiency can be improved by increasing sample pooling and template production through approaches such as COLD-PCR and QMC-PCR. Technical advances developed to improve clinical diagnostics can play a role in the targeted mutation breeding of crops.

## Background

Crops with improved traits are being developed by screening for mutations induced in candidate genes [1-5]. Several methods have been used to screen plant populations mutagenized by chemicals such as ethyl methanesulfonate (EMS). EMS-mutagenized tobacco lines, for example, were screened by SSCP analysis [1]. Tobacco genotypes with induced mutations in the nicotine N-demethylase gene (*NtabCYP82E4*) were identified that had dramatically reduced levels of nornicotine. TILLING was used to screen EMS-mutagenized lines of a wheat variety null for *Wx-B1*, one of three *waxy *homeologs involved in starch biosynthesis [2]. Wheat genotypes with induced *Wx-A1 *and W*x-D1 *mutations were detected and later crossed to produce *wx-a1/wx-b1/wx-d1 *grain with low amylose starch. A third mutation scanning method, high resolution melting (HRM), was used to identify tomato lines with EMS-induced mutations in candidate genes regulating fruit quality and drought tolerance [3].

Modifications that improve screening throughput and sensitivity would expedite the screening of thousands of genotypes for natural or induced mutations. High-throughput capillary electrophoresis, for example, has facilitated mutation analysis by SSCP [1] and TILLING [6,7]. The adaption of HRM for clinical screening of human disease genes has encouraged the development of improvements that make it more sensitive, user-friendly, and cost-efficient. We examined the application of two HRM modifications, COLD-PCR [8] and QMC-PCR [9], to mutation screening of plant genes.

One approach to increasing HRM throughput is through the pooling of samples for analysis. Gady *et al. *[3] found that tomato lines could be reliably analyzed by HRM in pools of four genotypes, but 8-fold pooling increased the frequency of false negatives. HRM analysis of EMS-mutagenized maize was conducted with 5-fold pooling [10]. HRM throughput can be important for medical diagnostics [*e.g. *11], but more often the issue is detecting mutations in cells that comprise a small fraction of an otherwise normal tissue sample [12]. Increasing HRM sensitivity would improve mutation analysis of heterogeneous tissue samples, as well as pooled individuals.

COLD-PCR is a PCR modification that increases the sensitivity of mutation screening by favoring the production of amplicons with a DNA mismatch [13]. PCR is carried out with a denaturation temperature at which heteroduplexed DNA is preferentially denatured and amplified. The sensitivity of mutation detection by Surveyor^®^, a mismatch-specific endonuclease used in TILLING, was increased by more than an order of magnitude through enrichment for variant sequences [13]. COLD-PCR has been used in conjunction with HRM to identify genetic mutations as low as 0.1% in a wild-type DNA background [8]. We examined whether COLD-PCR could be used to increase the sample pooling depth of HRM analysis.

Alternatively, the efficiency of mutation analysis could be increased by modifications in DNA template production from large populations. Techniques such as NEATTILL [14] and QMC-PCR [9] expedite DNA template preparation. QMC-PCR was developed to improve HRM analysis of mutations in DNA of formalin-fixed paraffin-embedded tissue, which is subject to DNA degradation and crosslinking. With QMC-PCR, an initial multiplex reaction produces templates that are used in secondary reactions with nested primers to amplify multiple regions per template. For detecting mutations in a background of wild-type DNA, QMC-PCR was demonstrated to be as sensitive as COLD-PCR and eight-fold more sensitive than Sanger sequencing [9]. To examine this approach, we tested the effect of genomic DNA template levels on HRM of an initial PCR amplicon and its product from a second PCR reaction with nested primers.

As an experimental system, we targeted two genes that regulate flowering in peach, *Prunus persica*. Peach orthologs of *AGAMOUS *(*PpAG*) and *TERMINAL FLOWER 1 *(*PpTFL1*) have been characterized and genomic sequence data are available [15-17]. The 2010 release of the draft genome sequence of peach (http://www.rosaceae.org) will facilitate new gene discovery. Functional and translational genomics in peach, however, are limited by its recalcitrance to genetic transformation. Peach is a candidate for targeted mutation breeding because of its compact diploid genome (220 Mbp), self-compatibility, and short juvenile stage (2-3 years) for a woody plant. In this study, peach cultivars from a southeastern US breeding program were screened by HRM for natural polymorphism in *PpAG *and *PpTFL1*. Using single-nucleotide polymorphisms (SNPs) identified in these genes, two approaches to improve HRM throughput were then examined: (1) increasing sample pooling and (2) using PCR products as templates for further PCR and HRM analysis.

## Results

### HRM detection of SNPs in peach floral genes

**Figure 1:**
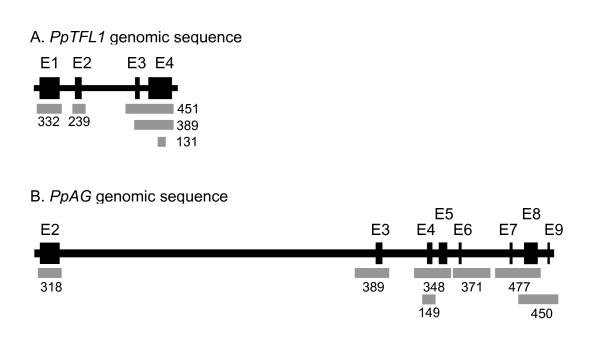
**Intron/exon structure of *PpTFL1 *(A) and *PpAG *(B)**. Exon 1 of *PpAG *is not translated and is not shown. Black boxes: exons; gray boxes: PCR amplicons, with length (bp).

**Figure 2:**
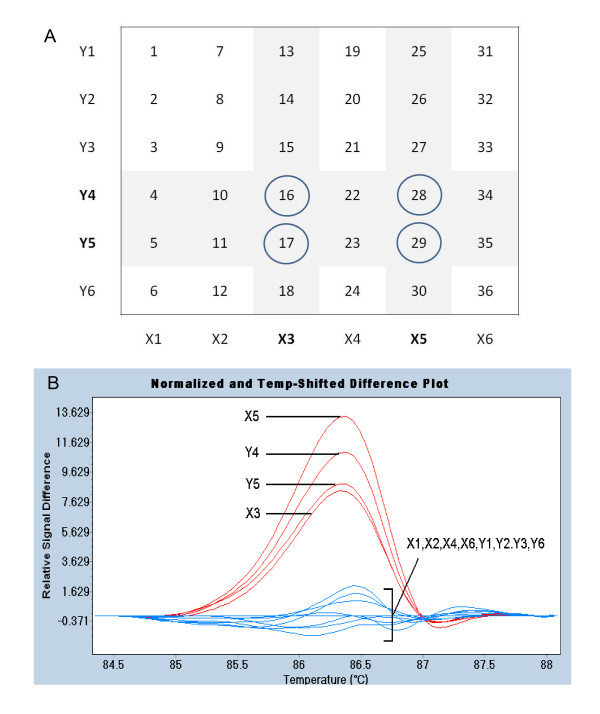
**HRM analysis of *PpTFL1 *exons 3 and 4**. A. Two-dimensional pooling of peach cultivars numbered 1-36. Pools of 6 cultivars were designated X1-X6 and Y1-Y6. Four pools (bold) with altered melting profiles have 4 cultivars in common (circled). B. HRM profile of pooled cultivars. Relative difference plot shows melting changes of pooled DNA compared to group X1.

Exons of *PpTFL1 *and *PpAG *were identified by alignment of genomic and cDNA sequences, and primer sets were developed that amplified exon regions (Figure 1). Genomic DNA isolated from 36 peach cultivars was pooled two-dimensionally in groups of six (Figure 2A). In addition to increasing throughput, sample pooling facilitated the detection of homozygous mutations by providing wild-type DNA for mismatch production. PCR and HRM were performed with a LightCycler 480 (Roche Diagnostics). The DNA melting data were analyzed by LC480 Gene Scanning software (version 1.5) which, after data normalization and temperature-shifting, grouped cultivars with similar melting patterns using a proprietary algorithm.

**Figure 3:**
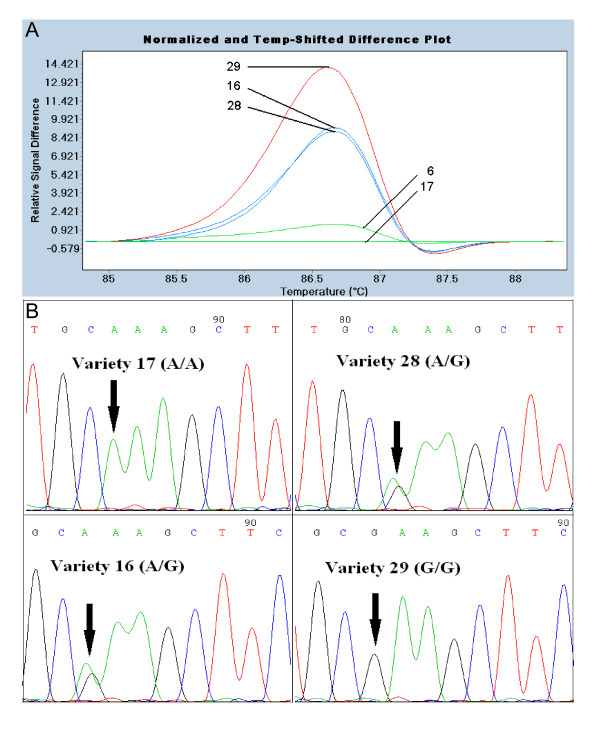
**Identification of cultivars containing SNPs in *PpTFL1 *exon 3 and 4**. A. Relative difference plot of cultivars 16, 17, 28, and 29. Each cultivar was mixed 1:1 with wild-type cultivar 6 to detect potential homozygous SNPs. Line colors indicate grouping by LC480 Gene Scanning software. B. Validation of SNPs by sequencing. Wild-type sequence (cultivar 17), homozygous SNP (cultivar 29), and heterozygous SNPs (cultivars 16, 28) at polymorphic site indicated by arrow.

HRM analysis of *PpTFL1 *exons 1 and 2 found no differences in DNA melting profiles among the 12 pools (not shown). In contrast, four pools exhibited altered DNA melting profiles when an amplicon spanning exons 3 and 4 was analyzed (Figure 2B). The four cultivars in common between these pools were examined independently, and three of them were found to have melting profiles that indicated a DNA mismatch (Figure 3A). DNA sequencing demonstrated that cultivars 16, 28, and 29 had a similar polymorphism (1202A > G) in *PpTFL1 *exon 4 and that cultivar 17 was wild-type (Figure 3B). Cultivar 29 had a homozygous SNP at this position, whereas cultivars 16 and 28 had heterozygous SNPs. Cultivar 29 grouped separately from the other SNP-containing lines due to a greater melting curve change likely caused by both *PpTFL1 *alleles forming mismatches at position 1202.

The DNA sequence of *PpTFL1 *exon 3 in cultivars 16, 17, 28, and 29 was found to be identical (not shown). The sequencing of *PpTFL1 *exons 3 and 4 in five other cultivars with wild-type HRM profiles found no polymorphism in this region (additional file 1). All pools without cultivars 16, 28, and 29 had similar wild-type HRM patterns for this region (Figure 2). Each *PpTFL1 *exon of all 36 peach cultivars was also examined individually by HRM and no SNPs were detected beyond those identified in pooled samples (additional file 2). These results show that a single polymorphic site (1202A > G) in the *PpTFL1 *coding region could be detected by HRM and that 3 of 36 cultivars contained this SNP.

**Figure 4:**
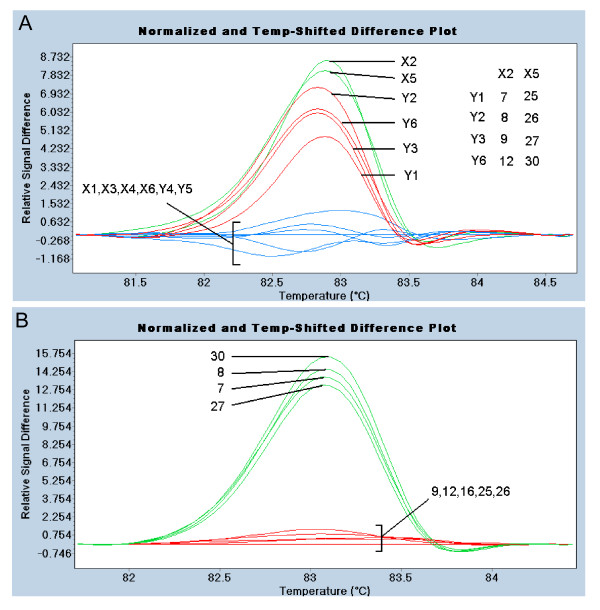
**HRM analysis of *PpAG *exons 4 and 5**. A. HRM profile of pooled cultivars. Relative difference plot shows melting changes of pools compared to group X1. Six pools with altered melting profiles have 8 cultivars in common (insert). B. Relative difference plot of cultivars 7, 8 9, 12, 25, 26, 27, and 30. Each cultivar was mixed 1:1 with wild-type cultivar 16 to detect potential homozygous SNPs.

The eight translated exons of *PpAg *were examined in six corresponding PCR amplicons ranging between 310-480 bp (Figure 1). Analysis of the amplicon spanning exons 4 and 5 identified six pools with altered melting profiles (Figure 4A), which contained 8 cultivars in common. When examined individually, four cultivars had melting profiles indicating a polymorphism (Figure 4B). This was confirmed by sequencing, which found that all four cultivars were heterozygous for a SNP in exon 4 (4757G > A). Pools containing two cultivars with the *PpAg *SNP (X2 and X5) grouped separately from pools with one SNP and no SNPs. The other five amplicons covering the *PpAG *coding region exhibited no DNA melting differences among the 36 cultivars (not shown). Table 1 summarizes the SNPs discovered in exons of *PpAG *and *PpTFL1*. No cultivar contained SNPs in both genes. For both genes, the SNPs resulted in synonymous mutations.

**Table 1:** SNPs identified in *PpTFL1 *and *PpAG*

Cultivar	*PpTFL1* nt position 1202	*PpAG* nt position 4757
consensus	A/A	G/G

# 7 Junegold	A/A	**A/G**

# 8 Juneprince	A/A	**A/G**

# 27 Flordaking	A/A	**A/G**

# 30 GulfCrest	A/A	**A/G**

# 16 Sunbrite	**A/G**	G/G

# 28 Flordadawn	**A/G**	G/G

# 29 Flordaprince	**G/G**	G/G

Corresponding nucleotides at the polymorphic site of each allele are shown. The consensus nucleotide at *PpTFL1 *position 1202 was based on sequenced amplicons of nine cultivars with melting profiles similar to 24 other cultivars. The consensus nucleotide at *PpAG *position 4757 was based on sequenced amplicons of three cultivars with melting profiles similar to 29 other cultivars.

### HRM analysis of pooled samples using standard PCR and COLD-PCR

**Figure 5:**
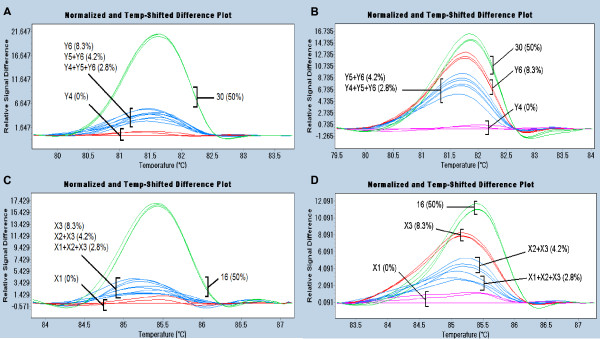
**Comparison of HRM of pooled samples after standard PCR or COLD-PCR**. A. Standard PCR/HRM analysis of a 131 bp amplicon from *PpAG *exon 4. Cultivar 30 was examined in pools of six (Y4), twelve (Y4 + Y5), and eighteen (Y4 + Y5 + Y6) genotypes. Percentage of SNP-bearing allele in pool is shown in parentheses. B. COLD-PCR/HRM analysis of a 131 bp amplicon from *PpAG *exon 4. C. Standard PCR/HRM analysis of a 149 bp amplicon from *PpTFL1 *exon 4. Cultivar 16 was examined in pools of six (X1), twelve (X1 + X2), and eighteen (X1 + X2 + X3) genotypes. D. COLD-PCR/HRM analysis of a 149 bp amplicon from *PpTFL1 *exon 4. Each pool was examined in triplicate. Line colors indicate grouping by LC480 Gene Scanning software.

Genotypes with polymorphisms in *PpAG *or *PpTFL1 *were detected in DNA pooled from six peach cultivars. We examined whether the SNPs could be identified in sample pools that were two or three times as large. Cultivar 30 (*PpAG *SNP) and cultivar 16 (*PpTFL1 *SNP) were each pooled in groups of 6, 12, or 18 genotypes with cultivars found to be wild-type for the gene examined. For both genes, the LC480 Gene Scanning software distinguished the three pools containing a SNP from a pool of cultivars with wild-type sequence (Figure 5A and 5C). However, the three SNP-containing pools were not distinguished from each other. Amplicons over 300 bp affected the repeatability of SNP detection at a 1:18 dilution, but not 1:6 or 1:12 dilutions (*e.g. *additional file 3). These data indicate that increasing the pool size to 12 genotypes is feasible in peach.

The use of COLD-PCR to preferentially amplify mismatched DNA was examined as a means to increase the sensitivity of HRM analysis of pooled samples. The T_m _of amplicons spanning the SNPs was determined by LC480 Gene Scanning software to be 85.7°C for *PpTFL1 *and 81.8°C for *PpAG*. The critical temperature (T_c_) for COLD-PCR was optimized using a range of denaturation temperatures approximately 1°C less than the T_m _of the amplicon. COLD-PCR with a T_c _of 84.5°C for *PpTFL1 *and 80.7°C for *PpAG *resulted in the enrichment of PCR amplicons with DNA mismatches (Figure 4 B and 4D). For both genes, the sensitivity of detection of SNPs in pooled samples increased relative to the SNP-containing cultivar alone (green lines). After COLD-PCR, the LC480 Gene Scanning software could distinguish the melting profile of SNPs in the 1:6 pool (red) from the larger pools. COLD-PCR results were consistent with amplicons of less than 150 bp (Figure 5B and 5D), but not with the amplicons over 300 bp that were tested (not shown).

### Effect of DNA template quantity and quality on HRM analysis

Two important features of QMC-PCR are (1) the production of initial PCR products from genomic template of varying availability and (2) the use of a resulting PCR product as template for analysis of multiple DNA regions with nested primers.

**Figure 6:**
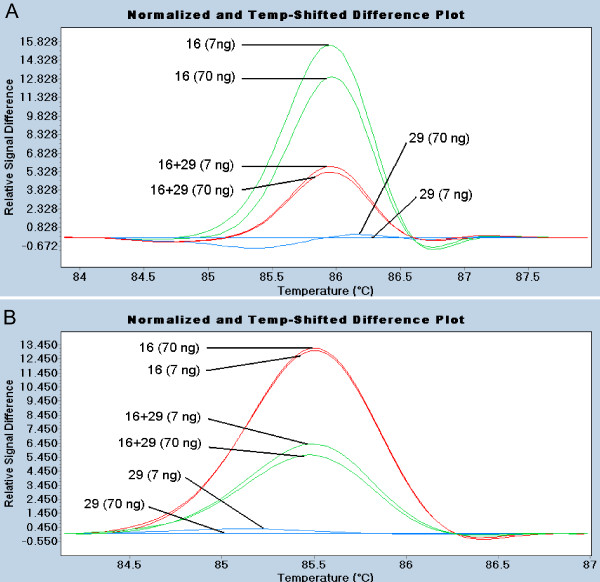
**HRM analysis of the SNP in exon 4 of *PpTFL1 *using varied level of genomic DNA templates**. A. Two levels (7 and 70 ng) of genomic DNA templates of cultivar 16, cultivar 29 and a 1:1 mixture of the two cultivars were used in a primary PCR amplification of exons 3+4. B. Diluted PCR products were used in the second round PCR using nested primers, producing internal amplicons of exon 4 which were analyzed by HRM.

A 10-fold difference in genomic template was first examined using genotypes with (cultivar 16) and without (cultivar 29) a SNP in *TFL1 *exon 4. HRM results were similar for template levels of 7 and 70 ng when these cultivars were analyzed separately and together (Figure 6A).

PCR products from the experiment described above were used as template for an internal region amplified with nested primers. The HRM results were similar to those obtained with genomic DNA, except that the melting temperature of the shorter amplicon was lower by approximately 0.5°C (Figure 6B). These results showed that a PCR product can be used as template for HRM and that a 10-fold difference in initial template concentration did not affect SNP detection. Techniques like QMC-PCR that mitigate variation in template quantity and quality could simplify DNA isolation from large plant populations.

## Discussion

HRM analysis of *PpTFL1 *and *PpAG *alleles of 36 peach cultivars found one polymorphic site in the coding region of each gene. Seven cultivars with SNPs were identified while screening 3374 bp of sequence per genotype. Wild-type melting profiles from individual and pooled samples corresponded with the sequencing results of 14 amplicons, making it unlikely that there are additional SNPs in *PpTFL1 *and *PpAG *exons. A comparison of the complete *PpTFL1 *genomic sequence of peach cultivars Lovell and Nemared found an SSR variation in intron 1, but no polymorphism in exon sequence [17].

In contrast to peach, HRM analysis of 25 cultivars of almond (*Prunus dulcis*) detected numerous SNPs in coding sequences, with an average frequency of 1:157 bp [18]. In olive (*Olea europea*), an outcrossing species like almond, variation in a 307 bp region of *phyA *was examined by HRM [19]. Sixteen of 38 olive cultivars had SNPs at one or two polymorphic sites within this region. The low level of genetic variability observed in *PpTFL1 *and *PpAG *may be a consequence of self-compatibility in peach and the narrow genetic base of cultivars bred for the eastern US [20,21]. Additionally, there may be selection against coding region mutations in *PpTFL1 *and *PpAG*, which are single genes in peach [16,17].

*PpTFL1 *and *PpAG *SNPs were used to examine approaches to increase HRM throughput. Following standard PCR of DNA pools of twelve cultivars, HRM analysis consistently identified pools with a SNP-containing cultivar. This pool size is larger than previously reported pools of four or five genotypes [3,10], possibly due to differences in instrumentation or genome size. HRM can detect a variant sequence diluted in wild-type DNA at ratios up to 1:200 [12]. HRM sensitivity, however, is lower with pooled DNA from different individuals than for a variant sequence diluted with DNA from a single source [3].

COLD-PCR increased the sensitivity of HRM analysis of pooled samples for *PpTFL1 *and *PpAG *SNPs. After COLD-PCR, melting profiles of pooled samples more closely resembled the melting profile of an individual SNP-containing genotype, presumably through enrichment for the sequence variant. In dilution experiments, HRM with COLD-PCR exhibited detection limits below 1% [8]. In this study, variant sequences comprising 2.8% of the pooled DNA were detected, although sample pools of more than 18 genotypes were not examined. COLD-PCR may be more useful for genotyping than mutation scanning because of limitations on amplicon size. COLD-PCR has been licensed for medical diagnostics and further research [*e.g. *22] may broaden the applicability of the technique.

HRM results were consistent for nested products produced from PCR-derived template, despite 10-fold differences in genomic template in the original PCR reaction. This suggests that an approach like QMC-PCR could reduce the need for highly purified DNA from high throughput sample preparation. QMC-PCR captures variable levels of intact target regions in fixed archival tissue, where DNA degradation is problematic [9]. Dilution experiments with human DNA found that QMC-PCR could detect variant sequences present at 2.5% of a background of wild-type DNA.

In contrast to QMC-PCR and COLD-PCR, Sanger sequencing does not detect mutations present at less than 20% of total DNA [8,9]. Next-generation sequencing, though, has considerable potential as a mutation screening tool when strategies to distinguish mutations from sequencing errors are employed and sample pooling is used to improve cost-efficiency [23]. Roche 454 sequencing, for example, was used to identify EMS-mutagenized candidate genes in pooled samples of tomato [24] and petunia [25]. Direct comparisons of pyrosequencing and COLD-PCR or QMC-PCR-enhanced HRM found that the modified HRM analyses had an equal or lower limit of detection [26,27]. Diagnostic methods like HRM that detect mismatched DNA can be an alternative or complement to sequencing.

## Conclusions

Mutation scanning by HRM could identify SNPs in exons of *PpAG *and *PpTFL1 *in a small set of peach cultivars. Cultivars with SNPs in these genes were used to determine that polymorphisms could be reliably detected in pools of twelve genotypes. COLD-PCR was found to increase the sensitivity of HRM analysis of pooled samples, but worked best with small amplicons. Examination of another HRM modification, QMC-PCR, demonstrated that primary PCR products for further analysis could be produced from variable levels of genomic DNA, providing an approach for simplifying high-throughput DNA isolation. Technical advances developed to improve clinic-based mutation screening can play a role in the targeted mutation breeding of crops.

## Methods

### Gene sequences and primers

The *PpAG *genomic sequence (GenBank FJ184275) was from peach cultivar Redhaven and the *PpTFL1 *genomic sequence was from the cultivar Lovell [17]. The intron/exon structure for *PpAG *and *PpTFL1 *was determined by using the Spidey alignment program [28] to compare the genomic sequences with *PpAG *mRNA (GenBank AY705972) and *MdTFL1 *mRNA (GenBank AB366643), respectively. Beacon Designer 7 software (Premier Biosoft) was used to design oligonucleotide primers to amplify exon regions (additional file 4). The primers were synthesized and HPLC-purified by MWG Operon (Huntsville, AL).

### Genomic DNA isolation and PCR template preparation

Leaves of 36 peach cultivars (additional file 5) were collected at the USDA Southeastern Fruit and Tree Nut Research Laboratory (Byron, GA). Total DNA was isolated using the DNeasy Plant kit (Qiagen) and quantified with a NanoDrop 800 spectrophotometer (Thermo Scientific). A total of 30 ng DNA was used for PCR, either from individual cultivars or sample pools. Primary pools of six cultivars were combined to test larger pool sizes of 12 and 18 cultivars. For QMC-PCR experiments, 7 ng or 70 ng of genomic DNA from cultivars 16 and 29 was used in PCR reactions with primers TE3MF and TE4R. To test the use of PCR product as template, the amplicons from these reactions were diluted 1:100 in ddH_2_O, and 2 μl of the dilution was used to amplify an internal fragment with primers TE4F and TE4R.

### PCR and COLD-PCR

PCR were carried out with a Mastercycler (Eppendorf) in reaction volumes of 20 μl containing 30 ng DNA, 0.2 μM of each primer, 2.5 mM MgCl_2_, and 2X Roche HRM Master Mix (with ResoLight dye). Reactions were denatured at 95°C for 3 minutes, followed by 45 cycles of 95°C for 20s, 55°C for 20s, 72°C for 30s, and a final extension at 72°C for 5 minutes. COLD-PCR was conducted with cultivars 16 or 30 in pools containing other cultivars known to be wild-type. For the *pTFL1 *exon 4 SNP analysis, this included pools X3, X3 + X2, and X3 + X2 + X1 (Figure 1A). Analysis of the *PpAG *exon 4 SNP used pools Y6, Y6+Y5, and Y6+Y5+Y4. Conditions for COLD-PCR of *pTFL1 *exon 4 were: 95°C for 3 minutes; 20 cycles of 95°C for 20s, 55°C for 20s, 72°C for 30s; heteroduplex formation through 94° for 30 seconds and 70°C for 8 minutes; and 20 cycles of 84.5°C for 20s, 61°C for 20s, and 72°C for 25s. Conditions for COLD-PCR of *PpAG *exon 4 were similar except that the final 20 cycles were: 80.7°C for 20s, 52°C for 20s, and 72°C for 25s.

### High resolution melting analysis and amplicon sequencing

On a LightCycler 480 (Roche Diagnostics), PCR products were denatured at 95°C for 1 minute, cooled to 40°C for 1 minute, and then heated to 95°C at 0.02°C/second, while continuously measuring florescence with 25 data acquisitions/°C. DNA melting data were analyzed by LC480 Gene Scanning software with settings for sensitivity and temperature shifting at 0.3 and 5, respectfully. All PCR/HRM experiments presented were repeated at least three times. For sequencing, PCR products were isolated by agarose gel electrophoresis and purified using a PureLink™ Quick Gel Extraction kit (Invitrogen). DNA samples were sequenced by MWG Operon (Huntsville, AL).

## Abbreviations

SSCP: single-strand conformation polymorphism; TILLING: targeting induced local lesions in genomes; COLD-PCR: co-amplification at lower denaturation temperature-PCR; QMC-PCR: quick-multiplex-consensus-PCR; NEATTILL: nucleic acid extraction from arrayed tissue for TILLING

## Authors' contributions

YC designed and performed the experiments. YC and HDW analyzed the data. HDW conceived of the study. HDW and YC contributed to the manuscript preparation, and read and approved the final manuscript.

## Supplementary Material

Additional file 1 ***PpTFL1 *exon 4 sequence**. PCR products spanning *PpTFL1 *exons 3 and 4 were sequenced from 9 peach cultivars. Only the sequence flanking the polymorphic site (arrow) in exon 4 is shown; the remaining sequence was identical. PCR products from cultivars 16 and 29 were also subcloned before sequencing, allowing SNP-containing alleles to be identified. Click here for file

Additional file 2 **Individual HRM analysis of 36 peach cultivars**. PCR products spanning *PpTFL1 *exons 3 and 4 were amplified in separate reactions for each cultivar and analyzed by HRM. Cultivars 16, 28, and 29 demonstrated altered melting patterns when HRM was repeated, but cultivar 21 did not. Click here for file

Additional file 3 **HRM analysis of *PpAG *exons 4 + 5**. Cultivar 30 was examined in pools of six (Y4), twelve (Y4 + Y5), and eighteen (Y4 + Y5 + Y6) lines. A relative difference plot of melting profiles of a 348 bp amplicon spanning *PpAG *exons 4 and 5 is shown. Group designations refer to pooling strategy shown in Figure 2A. HRM analysis was performed in triplicate and line colors indicate grouping by LC480 Gene Scanning software. Replicates of 12-fold pools were consistently differentiated from the pool of wild-type lines (Y4), but 18-fold pools were not. Click here for file

Additional file 4 **PCR primers for amplification of *PpTFL1 *and *PpAG *exons**. Click here for file

Additional file 5 **Peach cultivars analyzed**. Click here for file
