# Supplementary material for: Mutation scanning of peach floral genes
Source: BMC Plant Biol. 2011 May 23;11:96. doi: 10.1186/1471-2229-11-96 (PMC3120741; doi:10.1186/1471-2229-11-96)
Supplement: Additional file 1 — PpTFL1 exon 4 sequence. PCR products spanning PpTFL1 exons 3 and 4 were sequenced from 9 peach cultivars. Only the sequence flanking the polymorphic site (arrow) in exon 4 is shown; the remaining sequence was identical. PCR products from cultivars 16 and 29 were also subcloned before sequencing, allowing SNP-containing alleles to be identified. [file 1471-2229-11-96-S1.PDF]

```
                                          ↓
TFL1_exon4_P29_plasmid     TCAGCTGCGAAGCTTCGAGCACTGAAATGATCCCTTGAGGAAGGAGGGTT 249
TFL1_exon4_P4              TCAGCTGCAAAGCTTCGAGCACTGAAATGATCCCTTGAGGAAGGAGGGTT 149
TFL1_exon4_P16             TCAGCTGCAAAGCTTCGAGCACTGAAATGATCCCTTGAGGAAGGAGGGTT 150
TFL1_exon4_P17             TCAGCTGCAAAGCTTCGAGCACTGAAATGATCCCTTGAGGAAGGAGGGTT 150
TFL1_exon4_P1              TCAGCTGCAAAGCTTCGAGCACTGAAATGATCCCTTGAGGAAGGAGGGTT 143
TFL1_exon4_P20             TCAGCTGCAAAGCTTCGAGCACTGAAATGATCCCTTGAGGAAGGAGGGTT 143
TFL1_exon4_P8              TCAGCTGCAAAGCTTCGAGCACTGAAATGATCCCTTGAGGAAGGAGGGTT 142
TFL1_exon4_P29             TCAGCTGCGAAGCTTCGAGCACTGAAATGATCCCTTGAGGAAGGAGGGTT 106
TFL1_exon4_P22             TCAGCTGCAAAGCTTCGAGCACTGAAATGATCCCTTGAGGAAGGAGGGTT 102
TFL1_exon4_P28             TCAGCTGCAAAGCTTCGAGCACTGAAATGATCCCTTGAGGAAGGAGGGTT 105
TFL1_exon4_P16_plasmid     TCAGCTGCGAAGCTTCGAGCACTGAAATGATCCCTTGAGGAAGGAGGGTT 148
                           ******** *****************************************
```

Additional file 1- *PpTFL1* exon 4 sequence. PCR products spanning *PpTFL1* exons 3 and 4 were sequenced from 9 peach cultivars. Only the sequence flanking the polymorphic site (arrow) in exon 4 is shown; the remaining sequence was identical. PCR products from cultivars 16 and 29 were also subcloned before sequencing, allowing SNP-containing alleles to be identified.
