# Supplementary material for: Mutation scanning of peach floral genes
Source: BMC Plant Biol. 2011 May 23;11:96. doi: 10.1186/1471-2229-11-96 (PMC3120741; doi:10.1186/1471-2229-11-96)
Supplement: Additional file 2 — Individual HRM analysis of 36 peach cultivars. PCR products spanning PpTFL1 exons 3 and 4 were amplified in separate reactions for each cultivar and analyzed by HRM. Cultivars 16, 28, and 29 demonstrated altered melting patterns when HRM was repeated, but cultivar 21 did not. [file 1471-2229-11-96-S2.PDF]

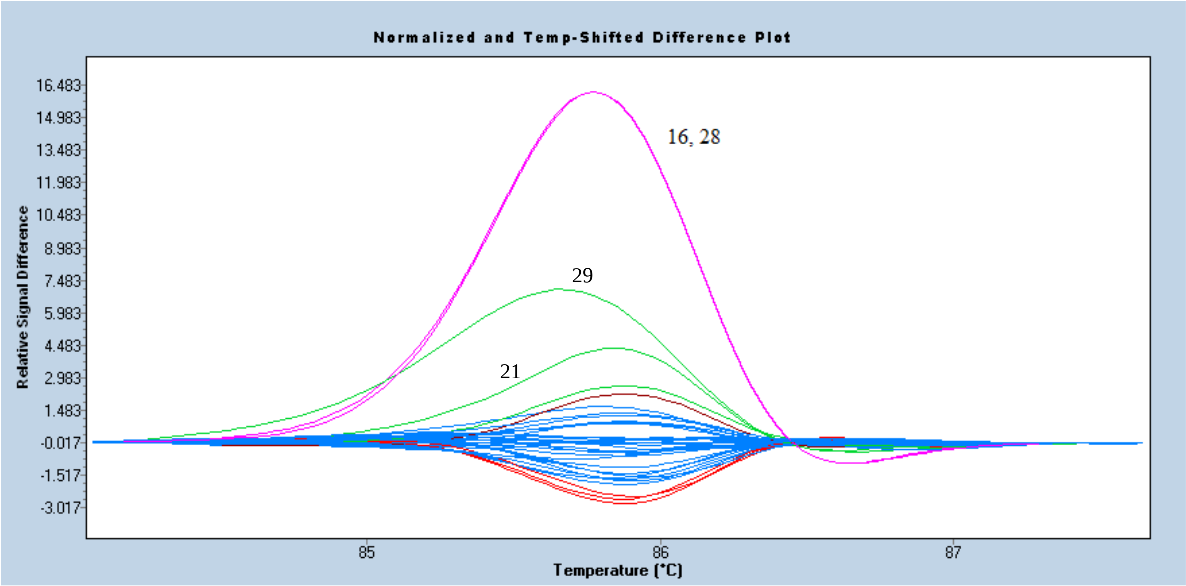

Additional file 2- Individual HRM analysis of 36 peach cultivars. PCR products spanning *PpTFL1* exons 3 and 4 were amplified in separate reactions for each cultivar and analyzed by HRM. Cultivars 16, 28, and 29 demonstrated altered melting patterns when HRM was repeated, but cultivar 21 did not.
