# Supplementary material for: Mutation scanning of peach floral genes
Source: BMC Plant Biol. 2011 May 23;11:96. doi: 10.1186/1471-2229-11-96 (PMC3120741; doi:10.1186/1471-2229-11-96)
Supplement: Additional file 3 — HRM analysis of PpAG exons 4 + 5. Cultivar 30 was examined in pools of six (Y4), twelve (Y4 + Y5), and eighteen (Y4 + Y5 + Y6) lines. A relative difference plot of melting profiles of a 348 bp amplicon spanning PpAG exons 4 and 5 is shown. Group designations refer to pooling strategy shown in Figure 2A. HRM analysis was performed in triplicate and line colors indicate grouping by LC480 Gene Scanning software. Replicates of 12-fold pools were consistently differentiated from the pool of wild-type lines (Y4), but 18-fold pools were not. [file 1471-2229-11-96-S3.PDF]

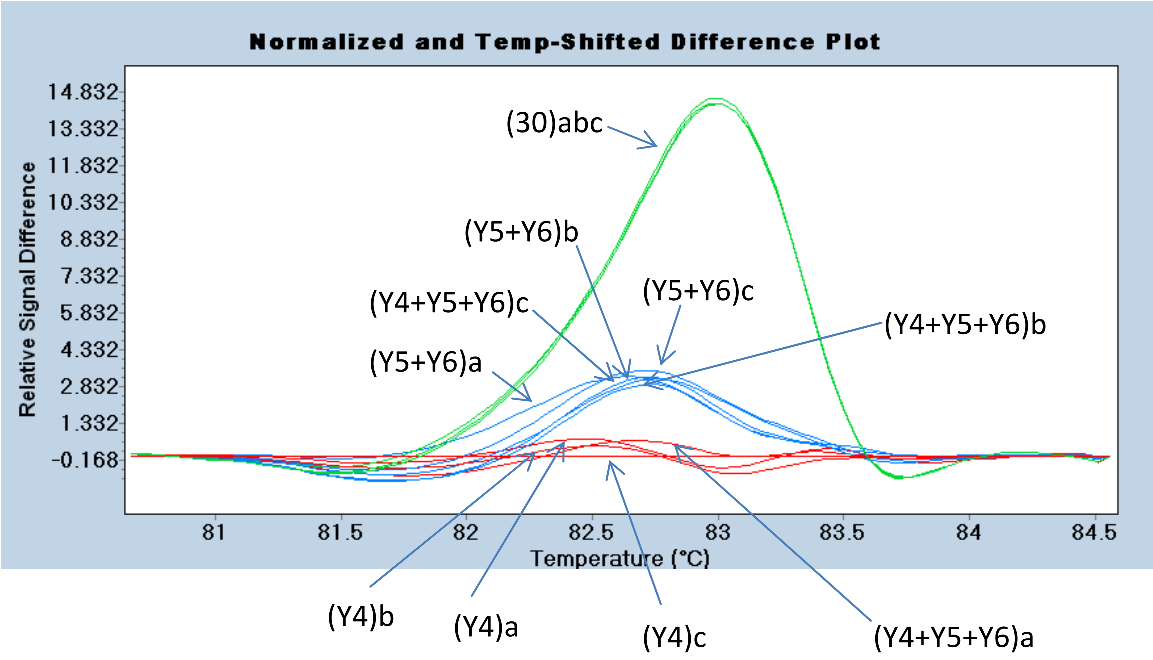

Additional file 3- HRM analysis of *PpAG* exons 4+5. Cultivar 30 was examined in pools of six (Y4), twelve (Y4+Y5), and eighteen (Y4+Y5+Y6) lines. A relative difference plot of melting profiles of the 348 bp amplicon spanning *PpAG* exons 4 and 5 is shown. Group designations refer to pooling strategy shown in Figure 2A. HRM analysis was performed in triplicate and line colors indicate grouping by LC480 Gene Scanning software. Replicates of 12-fold pools were consistently differentiated from the pool of wild-type lines (Y4), but 18-fold pools were not.
