# Supplementary material for: Mutation scanning of peach floral genes
Source: BMC Plant Biol. 2011 May 23;11:96. doi: 10.1186/1471-2229-11-96 (PMC3120741; doi:10.1186/1471-2229-11-96)
Supplement: Additional file 4 — PCR primers for amplification of PpTFL1 and PpAG exons. [file 1471-2229-11-96-S4.PDF]

Additional file 4- PCR primers for amplification of *PpTFL1* and *PpAG* exons

Name	Sequence (5’-3’)	Amplicon	Length (bp)	Amplicon location
**TE1MF**	CACTCCTCCTCATTCTCTCTGTCTC	**TE1MF-TE1JR**	**332**	***PpTFL1* exon 1**
**TE1JR**	TGATCCGATAATGAAGACTC			
**TE2MF**	ATTTTACTTTGCATCTCTTGTGA	**TE2F-TE2R**	**239**	***PpTFL1* exon 2**
**TE2R**	TGAAGAGGAGAAAGGGGGAGTACATC			
**TE3MF**	GCTAAAAGTTGTCTAAGTATATAA	**TE3MF-TE4R**	**451**	***PpTFL1* exons 3+4**
**TE4F**	TGAACTAAACCCAGGATTGTGACAGAT			
**TE4R**	GAGGAGCTTTGGGGTTTTGGGTAG	**TE4F-TE4R**	**389**	***PpTFL1* exon 4**
**T131F**	CAAGCAGACAAGAAGGCAGT	**T131F-T131R**	**131**	***PpTFL1* exon 4**
**T131R**	CCGTTTCTCTCTGGCAATT			
**AGE1F**	TTAGCTCCTTGATTGATCTCTTTG	**AGE1F-AGE1R**	**318**	***PpAG* exon 2**
**AGE1R**	AGAAATCGAAAAGAAAATAAAAGAAG			
**AGE2F**	AGCCCACTTACAGCATTTGAGG	**AGE2F-AGE2R**	**389**	***PpAG* exon 3**
**AGE2R**	GCTAATCTCGCTTTCAAAATCATACTC			
**AGE34F**	GCAGTGTTTATTGATTTTGA	**AGE34F-AGE34R**	**348**	***PpAG* exons 4+5**
**AGE34R**	ATCCATCATAAATATAGAAGAG			
**A149F**	CAATGAGATGTTTTAACC	**A149F-A149R**	**149**	***PpAG* exons 4**
**A149R**	AAGATTTTGGTAACTAAG			
**AGE5F**	TTTTTGCGATCTGAATTATTTACTTGT	**AGE5F-AGE5R**	**371**	***PpAG* exon 6**
**AGE5R**	GCCTACAGTCTCCAAATGCATATG			
**AGE67F**	ATTCATATAAGTTTTGTTCT	**AGE67F-AGE67R**	**477**	***PpAG* exons 7+8**
**AGE67R**	GATTTGTTATGAGGTTGA			
**AGE78F**	GGGGCTTGTGAATGGGATATATAG	**AGE78F-AGE78R**	**450**	***PpAG* exons 8+9**
**AGE78R**	TTGATGATTTATGCTTCCTACTACTTG			
