# Supplementary material for: Mutation scanning of peach floral genes
Source: BMC Plant Biol. 2011 May 23;11:96. doi: 10.1186/1471-2229-11-96 (PMC3120741; doi:10.1186/1471-2229-11-96)
Supplement: Additional file 5 — Peach cultivars analyzed. [file 1471-2229-11-96-S5.PDF]

Additional file 5- Peach cultivars analyzed

1. Redhaven
2. Blazeprince
3. Cresthaven
4. Gala
5. Goldprince
6. Jefferson
7. Junegold
8. Juneprince
9. Redglobe
10. Rubyprince
11. Springprince
12. Summergold
13. Sunland
14. Sunprince
15. Sureprince
16. Sunbrite
17. Fire Prince
18. Southern Pearl
19. Autumnprince
20. Augustprince
21. Big Red
22. Contender
23. Dixiland
24. Elberta
25. Fay Elberta
26. Flavorcrest
27. Flordaking
28. Flordadawn
29. Flordaprince
30. GulfCrest
31. Gulfprince
32. Harvester
33. Julyprince
34. Majestic
35. OHenry
36. Surecrop
